# Expression and characterization of duck enteritis virus gI gene

**DOI:** 10.1186/1743-422X-8-241

**Published:** 2011-05-19

**Authors:** Lijuan Li, Anchun Cheng, Mingshu Wang, Jun Xiang, Xiaoyuan Yang, Shunchuan Zhang, Dekang Zhu, Renyong Jia, Qihui Luo, Yi Zhou, Zhengli Chen, Xiaoyue Chen

**Affiliations:** 1 Institute of Preventive Veterinary Medicine, Sichuan Agricultural University, Wenjiang, Chengdu city, Sichuan, 611130, P.R.China; 2 Avian Disease Research Center, College of Veterinary Medicine of Sichuan Agricultural University, 46# Xinkang Road, Ya'an, Sichuan 625014, P.R. China; 3 Key Laboratory of Animal Disease and Human Health of Sichuan Province, Sichuan Agricultural University, Wenjiang, Chengdu city, Sichuan, 611130, P.R.China

## Abstract

**Background:**

At present, alphaherpesviruses gI gene and its encoding protein have been extensively studied. It is likely that gI protein and its homolog play similar roles in virions direct cell-to-cell spread of alphaherpesviruses. But, little is known about the characteristics of DEV gI gene. In this study, we expressed and presented the basic properties of the DEV gI protein.

**Results:**

The special 1221-bp fragment containing complete open reading frame(ORF) of duck enteritis virus(DEV) gI gene was extracted from plasmid pMD18-T-gI, and then cloned into prokaryotic expression vector pET-32a(+), resulting in pET-32a(+)-gI. After being confirmed by PCR, restriction endonuclease digestion and sequencing, pET-32a(+)-gI was transformed into *E.coli *BL21(DE3) competent cells for overexpression. DEV gI gene was successfully expressed by the addition of isopropyl-β-D-thiogalactopyranoside(IPTG). SDS-PAGE showed that the recombinant protein His6-tagged gI molecular weight was about 61 kDa. Subsequently, the expressed product was applied to generate specific antibody against gI protein. The specificity of the rabbit immuneserum was confirmed by its ability to react with the recombinant protein His6-tagged gI. In addition, real time-PCR was used to determine the the levels of the mRNA transcripts of gI gene, the results showed that the DEV gI gene was transcribed most abundantly during the late phase of infection. Furthermore, indirect immunofluorescence(IIF) was established to study the gI protein expression and localization in DEV-infected duck embryo fibroblasts (DEFs), the results confirmed that the protein was expressed and located in the cytoplasm of the infected cells, intensively.

**Conclusions:**

The recombinant prokaryotic expression vector of DEV gI gene was constructed successfully. The gI protein was successfully expressed by *E.coli *BL21(DE3) and maintained its antigenicity very well. The basic information of the transcription and intracellular localization of gI gene were presented, that would be helpful to assess the possible role of DEV gI gene. The research will provide useful clues for further functional analysis of DEV gI gene.

## Background

Duck virus enteritis(DVE), also called duck plague, is an acute and contagious herpesvirus infection of waterfowls such as ducks, geese, and swans with high morbidity and mortality[1]. The causative agent of DVE is duck enteritis virus (DEV), which is a member of subfamily *Alphaherpesvirinae *of the family *Herpesviridae*, not assigned to any genus according to the Eighth International Committee on Taxonomy of Viruses (ICTV)[2]. Like other herpesvirus, DEV establishes a lifelong infection, via a quiescent state known as latency. The genome of DEV is composed of a linear, double stranded DNA and the G+C content is 64.3%, higher than any other reported avian herpesvirus in the subfamily *Alphaherpesvirinae*[3]. Recently, an increasing number of DEV genes, such as UL5[4], UL6[5], UL22, UL23(TK)[6], UL24[6,7], UL25-UL30[8], UL31-UL35[9-11], UL38[12], UL44(gC)[13], UL46[14], UL50(dUTPase)[15], UL51[16], UL53(gK)[17], US3-US5[18,19], US8(gE)[20], US2 and US10[21], have been identified. The DEV genomic library was successfully constructed in our laboratory [22], and the gI(Us7) gene(GenBank accession no.: EU035298) was isolated and identified from DEV CHv strain[23].

The gI gene is located in unique short region (Us) within the herpesviral genome, its homolog almost existed in all alphaherpesvirus. The gI gene encoding membrane protein glycoprotein I(gI) is conserved among the alphaherpesviruses that have been sequenced. At present, the most extensively studied on alphaherpesviruses gI gene and its encoding protein are herpes simplex virus type 1(HSV-1), varicella-zoster virus(VZV), and pseudorabies virus(PRV). In all instances studied to date, the glycoprotein I (gI) and glycoprotein E (gE) form a noncovalent complex gE/gI that are localized to the plasma membrane, the virion envelope, and all internal membranes (except for mitochondria) in infected cells[24]. Biological functions ascribed to gE/gI include cell-cell spread, binding of antibody immunoglobulin G (IgG) Fc receptor. Alphaherpesvirus gI protein played an important role in virion sorting and promoting direct cell-to-cell spread in polarized cells, but not enrty of extrcellular virions[25]. Moreover, gI complexed with gE in HSV-1[26], VZV[27] and PRV[28] to form Fc-receptor, participating in immune escape. Previous sequence analysis of DEV CHv strain gI gene indicated that the ORF was 1116 bp in length and its primary translation product was a polypeptide of 371 amino acids. The predicted protein possessed several characteristics of membrane glycoproteins and had a high degree of similarity to gI homologs of other alphaherpesviruses[23]. Comparison of predicted amino acid sequences to those of HSV-1, VZV, and PRV homologs allowed the functions of DEV gI protein to be putatively assigned. Nevertheless, little is known about the characteristics of DEV gI gene.

In our study, the gI gene of DEV CHv-strain was extract from recombinant plasmid pMD18-T-gI, in an effort to elucidate the function of gI, we constructed a recombinant plasmid pET-32a(+)-gI and successfully expressed the DEV gI fused to His6 in a prokaryotic expression system. We prepared polyclonal antiserum which allowed identifying and characterizing the gI gene product of DEV. The levels of the mRNA transcripts of gI were determined by a real time-PCR method. In addition, the primary antibody against the DEV gI recombinant protein was used for intracellular localization by an indirect immunofluorescence assay(IIF). Taken together, the results indicate that the gI gene was transcribed most abundantly during late phase of infection, and the protein was expressed in DEV-infected DEFs, principally locating in cytoplasm of the infected cells. This work may provide a foundation for further studies on the function of DEV gI gene.

## Results

### Identification of recombinant plasmid

**Figure 1:**
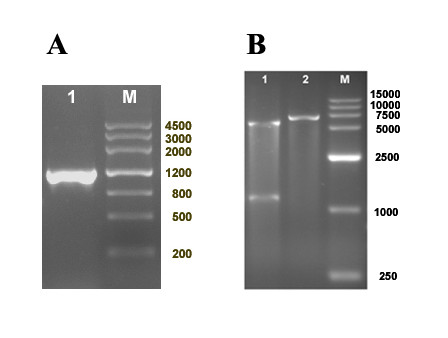
**Identification of the recombination vector pET-32a(+)-gI**. **A**. PCR product of the fragment of DEV gI detected by 1% agarose gel electrophoresis. Lane M, DNA marker(in bp); Lane 1, PCR product of the DEV gI. **B**. DEV gI gene encoding DNA sequence was cloned into pET-32a(+) prolaryotic expression vector as described in materials and methods. The construct was digested with two restriction enzymes. Lane M(in bp), DNA marker; Lane 1, *Bam*HI and *Xho*Ι generating two restriction fragments; Lane 2, *Bam*HI generating one restriction fragment.

The special 1221-bp fragment containing complete ORF of DEV gI gene was cloned into pET-32a(+) vector, resulting in construct pET-32a(+)-gI. For confirmation, plasmid DNAs of constructs was verified by PCR analysis(Figure 1A) and restriction enzyme digestion with *Bam*HI and *Xho*I(Figure 1B).

### Expression and purification of recombinant protein His6-tagged gI

**Figure 2:**
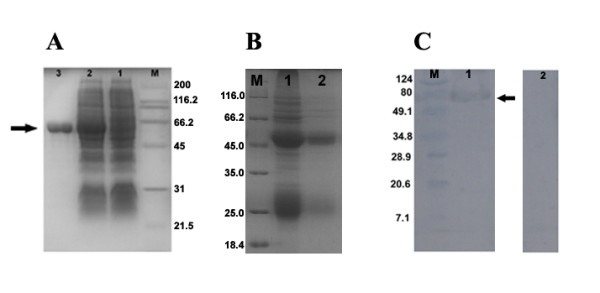
**SDS-PAGE of the purified fusion protein and rabbit anti-His6-tagged gI IgG and Western blotting analysis**. **A**. Induction of the His6-tagged gI fusion protien in *E. coli*. Recombinant plasmid pET-32a(+)-gI was transformed into bacteria. Lane M, molecular mass markers (in kDa); lane 1, protein products of the uninduced recombinant bateria; lane 2, protein products of the induced recombinant bateria; lane3, purified fusion protein His6-tagged gI. The arrowhead indicates the induced gI fusion protein; **B**. SDS-PAGE analysis of the purified rabbit anti-His6-tagged gI IgG. Lane M, protein molecular weight marker(in kDa); lane 1, rabbit anti-His6-tagged gI IgG obtained by ammonium sulfate precipitation; lane 2, rabbit anti-His6-tagged gI IgG obtained by High-Q anion exchange chromatography; **C**. Reactivity and specificity of the purified gI antiserum analyzed by Western blotting. Proteins were separated by SDS-PAGE and transferred to PVDF membranes. Lane M, protein molecular weight marker(in kDa); lane 1, the membranes were incubated with the gI antiserum; lane 2, the membranes were incubated with preimmune rabbit serum. Arrowheads indicate the fusion protein His6-tagged gI.

The expression products collected at different culture periods were characterized by SDS-PAGE and Western blotting. The results showed that there was a specific band with a molecular weight of 61 kDa in crude cell extracts(Figure 2A, lane 2), that is consistent with the calculated molecular weight of the DEV gI protein. SDS-PAGE revealed that the recombinant protein was expressed efficiently and constantly in *E.coli *BL21(DE3) cells. The expression level peaked 6 h after induction with 0.2 mM IPTG. Based on the His6 tag present at its N-terminal end, the recombinant gI was purified by Ni-NTA affinity chromatography (Figure 2A, lane 3). The purified protein was identified by rabbit anti-DEV serum in Western blotting (data not shown).

### Preparation and specificity of anti-His6-tagged gI protein antiserum

The rabbit anti-His6-tagged gI IgG, with 55 kDa and 25 kDa of the heavy chain and the light chain, was firstly precipitated by ammonium sulfate precipitation (Figure 2B, lane 1) and then purified by High-Q anion exchange chromatography (Figure 2B, lane 2). Western blotting analysis showed that the purified His6-tagged gI was recognized by the rabbit anti-His6-tagged gI IgG and showed a specific band at 61 kDa, which is the expected size of the fusion protein (Figure 2C, lane 1). No positive signal was observed when using the pre-immune serum (Figure 2C, lane 2), indicating that the recombinant protein induced an immunological response and that the antiserum had a high level of specificity. Based upon these results, this antiserum was deemed suitable to characterize the structure, molecular mechanism and functional involvement of the gI protein in the DEV life cycle.

### Determination of mRNA expression of gI in infected cells

**Figure 3:**
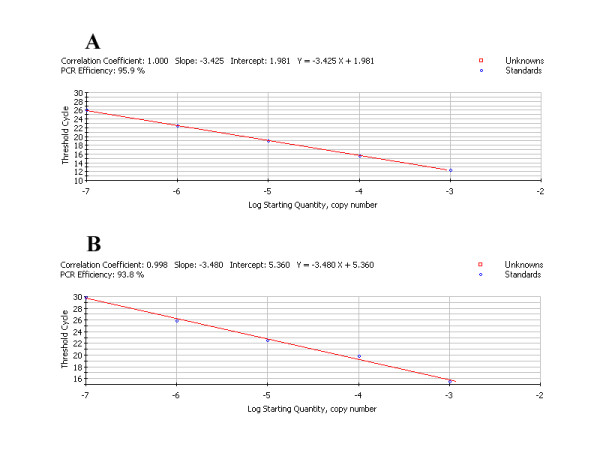
**Establishment of the real time-PCR(RT-PCR) standard curve**. **A**. The standard curve for gI gene obtained by SYBR Green RT-PCR using recombinant plasmid pMD18-T-gI as template. **B**. The standard curve for β-actin gene obtained by SYBR Green RT-PCR using recombinant plasmid pMD18-T-β-actin. Ten-fold dilutions of standard DNA were used, as indicated in the x-axis, whereas the corresponding cycle threshold (C_T_) values are presented on the y-axis. Each dot represents the result of amplification of each dilution. The correlation coefficient and the slope value of the regression curve were calculated and are indicated.

In the real time-PCR(RT-PCR) analysis, the dissociation curve of gI gene or β-actin gene showed a single peak at expected temperature(data not shown), that indicated specific amplification of those two genes. The standard curve(Figure 3) for gI and corresponding internal control β-actin gene obtained by RT-PCR using plasmid DNA as template showed similar correlation coefficient and PCR efficiency, it could be known that standard curve and the established RT-PCR are excellent at performance[29].

**Figure 4:**
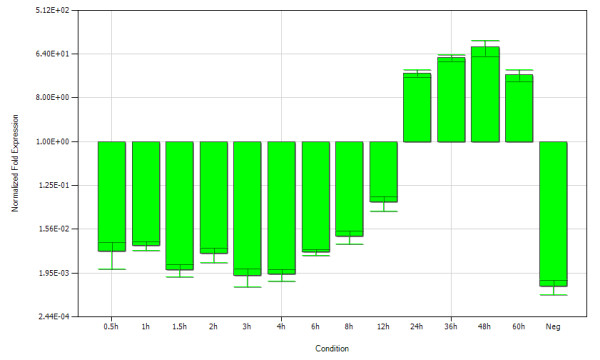
**Determination of mRNA expression of gI in DEV-infected DEFs**. The total RNA were harvested from uninfected(Neg) or DEV-infected DEFs at different times p.i. (0.5, 1, 1.5, 2, 3, 4, 6, 8, 12, 24, 36, 48, and 60 h), presented on the x-axis. After reverse transcription, the cDNA was used as template for SYBR Green RT-PCR analysis, the data were normalized to the expression level of β-actin, as indicated in the y-axis. Data were analyzed using the iQ5 optical system software (Bio-Rad)

The mRNA expression of gI gene at different times after infection were determined by normalizing the cycle threshold (C_T_) values with β-actin gene C_T _values, and then the histogram reflecting the transcription tendency of gI gene was constructed by iQ5 Optical System Software(Figure 4). It cound be found that, the level of mRNA was low in early phases of infection, presenting slightly increased after 3 h p.i.. Subsequently, signal intensity immediately increased after 12 h p.i., peaked at 48 h p.i., and then declined.

### Intracellular localization of the gI protein in DEV-infected cells

**Figure 5:**
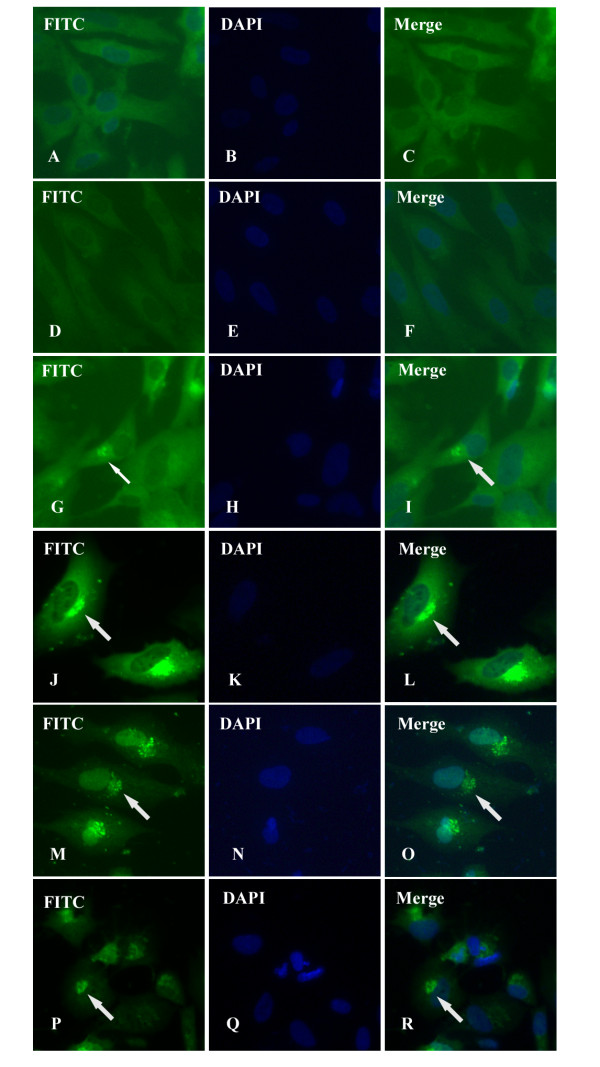
**Intracellular location and distribution of DEV gI proein analyzed by IIF**. Mock-infected (A-C) and DEV-infected (D-R). DEFs were fixed at different stages (4 h p.i., 12 h p.i., 36 h p.i., and 48 h p.i.) as described in materials and methods. The samples were stained with the gI antiserum (A-C, G-R) or preimmune serum (D-F), and reacted with anti-rabbit IgG-conjugated FITC, and then counter-stained with DAPI (blue is representative the cell nuclei). A, D, G, J, M, and P show the FITC staining; and B, E, H, J, M, and Q show the DAPI staining. C, F, I, L, O, and R are the merged images of FITC and DAPI staining. The arrows indicate the DEV gI FITC fluorescence staining. (Images were acquired by using 20× objective)

Intracellular distribution of DEV gI protein could be visualized by IIF experiments utilizing rabbit immune serum against expressed gI protein or pre-immune serum. As shown in Figure 5, infected cells (Figure 5G-R) showed a specific green fluorescent cytoplasmic staining pattern, whereas essentially no signal was detected in mock-infected cells(Figure 5A-C) or corresponding preimmune serum (Figure 5D-F). The faint fluorescence could be detected in the cytoplasm of infected cells as early as 4 h p.i. (Figure 5G-I), and then a strong fluorescence was found intensively distributed in the cytoplasm and especially in the juxtanuclear region at 12 h p.i.. A typical pattern of staining is shown in Figure 5J-L. After that, following by a series of morphological changes, the cytoplasm disintegration and nuclear fragmentation in DEV-infected cells, fluorescence was gently dispersed at 36 h p.i.(Figure 5M-O) and 48 h p.i. (Figure 5P-R).

## Discussion

Currently, gI gene has been studied extensively in human and nonhuman herpesviruses[27,30-33]. As mention in instruction, gI and gE formed a heterodimer gE/gI in alphaherpesviruses, gE/gI can promote direct cell-to-cell spread in polarized cells, but not entry of extracellular virions. Given that gE/gI specifically functions, this glycoprotein provides an excellent molecular tool to study cell-to-cell spread[34]. According to the previous report[23], a gene equivalent to the gI of other alphaherpesviruses was identified and sequenced in DEV CHv strain. The predicted amino acid sequence possesses several characteristics typical of membrane glycoproteins, including a N-terminal hydrophobic signal sequence, C-terminal transmembrane and cytoplasmic domains, and extra-cellular region containing three potential N-linked glycosylation sites. Compared with other alphaherpesviruses, DEV gI showed high identity at the amino acid level. But the analysis of its expression and characteristics have not been reported until now. Experimental determination of the DEV gI gene expression and localization in infected cells has become necessary.

The analysis of gene expression requires sensitive, precise, and reproducible measurement of specific mRNA sequences. The methods used to quantify mRNA include techniques based upon hybridization and real-time PCR(RT-PCR), RT-PCR is becoming a common tool for detecting and quantifying expression profiles of selected genes[35]. SYBR Green I is the most frequently used dsDNA-specific dye in RT-PCR today[36]. We have developed a rapid real-time quantitative PCR method using the icycler IQ Real-time PCR Detection System coupled with SYBR Green chemistry, to evaluate the time course of mRNA formation and decay of DEV gI gene. Recently, relative quantitation has become the analytic method of choice for many real-time PCR studies. In this method a comparison within a sample is made with the gene of interest to that of a control gene. Relative quantitation relies on the assumption that the endogenous control gene does not vary under the experimental conditions. Control genes that have been successfully used include β-actin, GAPDH, 18S ribosomal RNA, Histone 3.3a, ubiquitin, and several others[29]. In our study, to control for the variation in sample processing and in reverse transcription reaction among samples, DEF β-actin gene was amplified in parallel with the DEV gI gene. The chosen control gene β-actin does not vary in expression level among the samples of study.

Base on analyses of the HSV kinetics, both synthesis of virus proteins and transcription of virus DNA were coordinately regulated and sequentially ordered[37,38]. However, research on the expression kinetics of DEV genes has been rare. Our study showed that the gI gene of DEV transcription products appeared low level before 12 h p.i., then increased acutely and reached a peak at 48 h p.i., declining slowly thereafter, which owes the characterization of herpervirus late genes. Although gI gene of DEV was presumed as a late gene, its transcripts was keeping slightly increasing in the early phase of infection, that may relate to selective sorting of enveloped particles to cell junctions, the role gI played in the trans-Golgi network (TGN). After 12 h p.i., the transcription of gI gene sharply increased, compared with previous research, which revealed that DEV nucleocapsids first occurred at 12 h p.i., and mature viral with envelope first occurred at 23 h p.i in infected DEFs[39], it could be known that gI gene abundantly expressed when virion was enveloped, suggesting that the gene may be a late viral gene, which takes part in assembly with the envelope to form mature DEV virions. Thus, this study indicated that the determination of mRNA expression of gI gene in infected cells could provide critical clues for investing the gene characteristics and function, as well as the proliferation of virus.

Different intracellular localizations may reflect different functions of envelope proteins, e.g., it has been reported that, HSV gE/gI accumulated in the trans-Golgi network (TGN) at early times and then redistributed to cell junctions to promote cell-to-cell spread[40]. Numerous studies have demonstrated that gE/gI is targeted to the TGN or endosomes, sites where virus envelopment occurs. Furthermore, the accumulation of gE/gI depends on some sorting motifs in cytoplasmic domain of gE and gI, which are relate to cell-to-cell spread[41-44]. Although the intracellular localization of many alphaherpesvirus gI proteins, such as HSV-1, PRV, and VZV have been well characterized, we have only started to understand where DEV gI is targeted to. We characterized the intracellular localization of DEV gI by computer aided analysis[23] and IIF. Computer aided analysis suggested that DEV gI prodominantly located in the cytoplasm, similar to the homologous proteins of HSV-1[40,45], VZV[42], and Human cytomegalovirus(HCMV)[46], which were detected exclusively or predominantly in the cytoplasm. In this study, IIF analysis revealed that DEV gI intensively distribution in the cytoplasm, consistent with the computer prediction. According to our observations, DEV gI was detected as early as 4 h p.i. (Figure 5G-I), and then a strong fluorescence was observed mainly in the juxtanuclear region at 12 h p.i. (Figure 5J-L), probably associated with Golgi apparatus. Similarly, gE/gI accumulates predominately in the TGN at early times after HSV-1 infection(6 h p.i.), that appears to be important for virus assembly and as a first step towards the selective sorting of enveloped particles to cell junctions[47,48]. As proteins must be localized in the same intracellular compartment to co-operate towards a common biological function, we hypothesize that DEV gI serve some similar localization and functions of other alphaherpesvirus. However, further research is required to verify this hypothesis.

## Conclusions

In conclusion, the DEV gI gene has been successfully expressed in a prokaryotic expression system, and we prepared rabbit anti-His6-tagged gI serum with a high level of specificity. RT-PCR and IIF were used to study the expression and localization of gI gene. However, further studies involving the construction of a gI gene DEV mutant are required, which will reveal whether gI gene promotes cell-to-cell spread like other alphaherpesvirus. Moreover, to assess the functional cross-complementation of DEV gI gene and gE gene should also be important in further studies.

## Methods

### Cell and virus

DEV CHv strain, a high-virulence field strain, was isolated from the Key Laboratory of Animal Disease and Human Health of Sichuan Province. Duck embryo fibroblasts (DEFs) were cultured in Minimum Essential Medium (MEM) (Gibco-BRL) containing 10% fetal bovine serum (FBS) (Gibco-BRL) supplemented with 100 U of penicillin and 100 μg of streptomycin per ml. For DEV propagated in DEFs, MEM supplemented with 2% FBS was used.

### Plasmid construction

The full-length gI gene was designed to contain *Bam*HI and *Xho*I restriction sites and subcloned into pMD18-T vector (TaKaRa)[23]. The gI gene was digested with *Bam*HI and *Xho*I from the recombinant plasmid pMD18-T-gI, and then was purified using a TIANprep Mini Plasmid Kit (TianGen) according to the manufacturer's instructions. The purified products were cloned into prokaryotic vector pET-32a(+) (Novagen)subsequently. The recombinant plasmid pET-32a(+)-gI was confirmed by restriction enzyme digestion and PCR, the PCR steps were performed according to previous reports [23]. Sequencing reactions was performed by TaKaRa (Dalian, China).

### Prokaryotic expression and purification of recombinant protein His6-tagged gI

The recombinant plasmid pET-32a(+)-gI was transformed into *E.coli *BL21(DE3) competent cells according to the manufacturer's manual. A single colony of transformant was grown in Luria broth (LB) supplemented with 50 μg/ml ampicillin at 37°C until the OD600 reached 1.0. Then IPTG was added to a final concentration of 0.2 mM. The culture was incubated for an additional 6 h at 37°C. The cells were harvested by centrifugation and resuspended in 100 mM Tris-HCl (pH8.0). Cells were broken by sonication, insoluble material was collected by centrifugation at 10,000 × g for 10 min at 4°C, and solubilized proteins were analyzed by SDS-polyacrylamide gel electrophoresis (SDS-PAGE) followed by staining with coomassie brilliant blue. The expressed protein was further identified by recognition of rabbit anti-DEV antibody in Western blotting. His6-tagged proteins were purified by nickel affinity chromatography according to the manufacturer's protocol (Bio-Rad), and analyzed by SDS-PAGE.

### Preparation of polyclonal antibody against the recombinant protein

Each New Zealand white rabbit was injected three times at weekly intervals with 0.75 mg of purified recombinant protein His6-tagged gI mixed with an equal volume of Freund's complete adjuvant (Promega) on the back and proximal limbs. Subsequently, each rabbit was intravenously immunized with 0.05 mg of the purified recombinant protein. The animals were bled and the sera were harvested at two weeks after the final injection and stored at -70°C until further use. The purified IgG polyclonal antibodies were obtained by purification using ammonium sulfate precipitation [49] and High-Q anion exchange chromatography [50].

### Western blotting

To identify the specificity of the prepared antiserum,, Western blotting analysis was performed according to the standard procedure[51] using the purified rabbit anti-gI IgG. The proteins were separated by 12% SDS-PAGE and transferred by electroblotting onto polyvinylidene difluoride (PVDF) membrane according to the manufacturer's manual. The membrane was then blocked in 5% nonfat dry milk in PBS-T (0.5% Tween-20 in PBS, PH 7.4) for 1 h. After washing three times with PBS-T, the membrane was incubated with diluted rabbit anti-gI IgG or pre-immune serum (1:100) overnight at 4°C. Following three times washing with PBS-T, the membranes were incubated with horseradish peroxidase (HRP)-labeled goat anti-rabbit immunoglobulin G (IgG) (Zhongshan Co. Ltd., Beijing, China) at a dilution of 1:5000 for 1 h at 37°C. After three times washing with PBS-T, the membrane was reacted with 3,3'-diaminobenzidine (DAB) (Zhongshan Co. Ltd., Beijing, China) in the presence of 0.1% H_2_O_2_. The reaction was terminated by washing the membrane in distilled water.

### Determination of mRNA expression of gI in infected cells

The levels of the mRNA transcripts of gI were determined by a rapid real-time quantitative PCR(RT-PCR) method using icycler IQ Real-time PCR Detection System (Bio-Rad Corp., Hercules, CA) coupled with SYBR Green chemistry. SYBR Green dye has a high affinity for double-stranded DNA(ds-DNA) and exhibits enhancement of fluorescence upon binding to the dsDNA. The total RNA was extracted from uninfected or DEV-infected DEFs at different times (0.5 hr, 1 hr, 1.5 hr, 2 hr, 3 hr, 4 hr, 6 hr, 8 hr, 12 hr, 24 hr, 36 hr, 48 hr, and 60 hr postinfection [hp.i.]), using the Total RNA Isolation System(TaKaRa). The RNA integrity was assessed by running the samples in a 1% agarose gel following standard protocol. The concentration of RNA was determined by measuring A260, and the purity was checked by the A260/A280 ratio (greater than 1.8). The purified RNA was treated with 2 units DNase at 37°C for 30 min followed by inactivation at 65°C for 15 min. 2 μg RNA was used as template for reverse transcription at 37°C for 1 h to synthesize cDNA in Quantscript RT Kit(TianGen) according to the manufacturer's instructions. The RT-PCR primers designed based on the sequence of gI and β-actin cDNA are: gI forward primer (P1) is (5'-GCCGTGGAAGACAGAC-3') and gI reverse primer (P2) is (5'-CCAAGACGAGGGCAATCA-3'); β-actin forward primer (P1) is (5'-CCGGGCATCGCTGACA-3') and β-actin forward primer (P1) is (5'-GGATTCA TCATACTCCTGCTTGCT-3'). The primers were checked by running a conventional PCR and the amplifications were analyzed for expected product by electrophoresis in 3% agarose gels, cDNA equivalent of 5 ng original RNA was used in PCR. The β-actin mRNA expression was determined using the same amount of cDNA as an RNA-competence control. The standard curves of the real time-PCR were generated by successive dilutions of recombinant plasmid pMD18-T-gI or pMD18-T-β-actin, respectively. The amplifications were carried out in a 96 well plate in a 20 μl reaction volume containing 9 μl of SYBR Green Real Master Mix(TianGen), 0.5 μl each of forward and reverse primers and 1 μl of the 1:10 diluted recombinant plasmid. The temperature profile for SYBR Green RT-PCR was 95°C 1 min followed by 45 cycles of 95°C 5 s, 60°C 20 s and 72°C 25 s. SYBR Green RT-PCR of unknown samples was performed in a 96 well plate using 1 μl of each of the cDNA for gI gene or β-actin gene following the reaction parameters as described above. Each sample had 3 replicates, both negative control and blank control were run along with the unknown samples. After a SYBR Green RT-PCR run, data acquisition and subsequent data analyses were done using the icycler IQ Real-time PCR Detection System and iQ5 Optical System Software (BioRad). Each cycle threshold (C_T_) value was determined by iQ5 optical system software, and normalized by the β-actin expression level.

### Intracellular localization of the gI protein in DEV-infected cells

DEFs, grown on coverslips in a six-well culture plate, were either mock infected or infected with DEV CHv strain. The cells were harvested at different times postinfection (2 h p.i., 4 h p.i., 8 h p.i., 12 h p.i., 24 h p.i., 36 h p.i., 48 h p.i., and72 h p.i.), and then they were fixed with 4% paraformaldehyde for 30 min at room temperature. After washing with PBS-T, the fixed cells were treated with PBS buffer containing 0.2% Triton X-100 for 15 min to increase the cellular membrane permeability. The coverslips were then blocked for 1 h in PBS containing 5% bovine serum albumin at 37°C. The cells were washed three times for 5 min in PBS-T, then incubated with purified rabbit polyclonal antibodies IgG (1:100 dilution) specific for recombinant proteins DEV gI or pre-immune serum at 4°C overnight, washed three times for 5 min in PBS-T, and then treated with fluorescein isothiocyanate (FITC)-conjugated goat anti-rabbit IgG (Zhongshan Co. Ltd., Beijing, China) for 1 h at 37°C. The cell nuclei were visualized by 4', 6-diamidino-2-phenylindole (DAPI) counterstaining (5 mg/ml; Zhongshan Co. Ltd., Beijing, China). Fluorescent images were examined under the Bio-Rad MRC 1024 imaging system.

## Competing interests

The authors declare that they have no competing interests.

## Authors' contributions

LJL carried out most of the experiments and wrote the manuscript. ACC and MSW critically revised the manuscript and the experiment design. JX, XYY, SCZ, DKZ, RYJ, QHL, YZ, ZLC, XYC helped with the experiment. All of the authors read and approved the final version of the manuscript.
